# Emerging Applications of Picture Archiving and Communication Systems and Their Impact on Research and Education: A Literature Review

**DOI:** 10.7759/cureus.65019

**Published:** 2024-07-21

**Authors:** Albert P Varghese, Shreya Naik, Syed Asrar Ul Haq Andrabi, Anurag Luharia, Suhas Tivaskar, Jubin John, Gaurav V Mishra, Ashish Uke, Sweta G Pisulkar, Mayur Wanjari

**Affiliations:** 1 Department of Radiology, Datta Meghe Institute of Higher Education and Research, Wardha, IND; 2 Department of Radiation Oncology, Datta Meghe Institute of Higher Education and Research, Wardha, IND; 3 Department of Prosthodontics and Crown and Bridge, Sharad Pawar Dental College, Wardha, IND; 4 Department of Research and Development, Jawaharlal Nehru Medical College, Datta Meghe Institute of Higher Education and Research, Wardha, IND

**Keywords:** medical communication, radiodiagnosis, networking, radiology, pacs

## Abstract

In recent times, technological advancements have remarkably improved picture archiving and communication system (PACS) capabilities beyond their conventional use in radiology departments. Researchers and instructors have started employing PACS functionalities to improve medical research processes, promote interdisciplinary collaborations, and facilitate learning. To illustrate this point further, the PACS enables researchers to handle and analyze huge amounts of imaging data with superior precision and speed, supporting innovative studies in areas like disease progression, treatment outcomes, and imaging modalities. Moreover, a PACS integrated with artificial intelligence (AI) algorithms leads to significant improvements in image processing, diagnostic accuracy, and personalized treatment, thus marking a new approach to medical imaging. The PACS supported by AI is mostly transformative since they allow for improved early disease detection capabilities as well as automated image processing and decision assistance, which increase diagnostic accuracy and clinical outcomes. Such systems can rapidly process large quantities of visual data with an accuracy rate surpassing earlier endeavors. In medical research, however, combining PACS with AI allows challenging imaging datasets to be examined, thereby making findings that were not previously possible. The capacity to combine imaging outcomes with clinical information is valuable for medical students and professionals in the field of education. They can access extensive medical image collections and case studies using PACS. This link is critical for teaching and learning as it allows students to interact with concrete events and improve their diagnostic accuracy in a controlled environment. This review discusses how the PACS affects educational courses and clinical outcomes based on the available literature. Our aim was not only to outline recent research or developments but also to present a comprehensive overview regarding the growing role played by PACS in the modern healthcare system and academics. Similarly, we look at the challenges and opportunities associated with the wide adoption of PACS, highlighting possible future areas of study or teaching methodologies. Issues such as data security, interoperability, and the need for defined protocols are included to give an exhaustive understanding of what PACS can and cannot do. Through this study, we stress PACS's revolutionary potential in advancing research methodology and educational practices, eventually contributing to enhanced patient care and knowledge dissemination in healthcare areas. The continual growth of PACS technology and its applications is expected to reshape the landscape of medical research and education, making it a vital component in the quest for medical excellence. By knowing the present trends and future potential, stakeholders in healthcare and education may better employ PACS to reach their objectives and boost overall results.

## Introduction and background

In recent times, technological advancements have remarkably improved the picture archiving and communication system (PACS) capabilities beyond their conventional use in radiology departments. Researchers and instructors have started employing PACS functionalities to improve medical research processes, promote interdisciplinary collaborations, and facilitate learning. To illustrate this point further, the PACS enables researchers to handle and analyze huge amounts of imaging data with superior precision and speed, supporting innovative studies in areas like disease progression, treatment outcomes, and imaging modalities. Moreover, PACS integrated with artificial intelligence (AI) algorithms lead to significant improvements in image processing, diagnostic accuracy, and personalized treatment, thus marking a new approach to medical imaging. The PACS supported by AI is mostly transformative since they allow for improved early disease detection capabilities, automated image processing, and decision assistance, which increase diagnostic accuracy and clinical outcomes. Such systems can rapidly process large quantities of visual data with an accuracy rate surpassing earlier endeavors. In medical research, however, combining PACS with AI allows challenging imaging datasets to be examined, thereby making findings that were not previously possible.

The capacity to combine imaging outcomes with clinical information is valuable for medical students and professionals in the field of education. They can access extensive medical image collections and case studies using PACS. This link is critical for teaching and learning as it allows students to interact with concrete events and improve their diagnostic accuracy in a controlled environment. This review discusses how PACS affects educational courses and clinical outcomes based on the available literature. Our aim was not only to outline recent research or developments but also to present a comprehensive overview regarding the growing role played by PACS in the modern healthcare system and academics. Similarly, we look at the challenges and opportunities associated with the wide adoption of PACS, highlighting possible future areas of study or teaching methodologies. Issues such as data security, interoperability, and the need for defined protocols are included to give an exhaustive understanding of what PACS can and cannot do. Through this study, we stress PACS's revolutionary potential in advancing research methodology and educational practices, eventually contributing to enhanced patient care and knowledge dissemination in healthcare areas. The continual growth of PACS technology and its applications is expected to reshape the landscape of medical research and education, making it a vital component in the quest for medical excellence. By knowing the present trends and future potential, stakeholders in healthcare and education may better employ PACS to reach their objectives and boost overall results.

## Review

A computer-based PACS has been developed over the past 15 years to provide a more cost-effective alternative to conventional film management systems [1,2]. The major clinical purpose of automated radiology departments is to transmit high-quality data to doctors promptly. Automation has been beneficial in cutting operational expenses within radiology units. The usefulness of a PACS can be established if it fulfils either or both of these objectives [3-5].

While the expansion of PACS is typically driven by clinical and commercial aims, it also significantly facilitates the dissemination of medical knowledge. However, the objectives of research and education must be carefully addressed, necessitating meticulous planning and design.

In addition to furnishing clinical PACS with appropriate coding schemes and the requisite communication, storage, and display technologies for transportation, film is historically the main teaching resource for image-based radiological studies. Learners can readily analyze videos and information about photographs directly and simply. The learning methods mirror those employed in professional capacities. In recent years, there has been a burgeoning interest in developing automated teaching files [6-8] and increasingly sophisticated teaching systems. An ordinary radiology information system database, which tracks "interesting occurrences," is a basic example of an automated teaching tool. Instead of storing image data on computers, instructors input identifying details about conditions that may be educational.

The simplest form of digital teaching tool is a compilation of examples, often accompanied by digitized (or digital) images. Typically, such case study applications focus on specific areas of interest (e.g., pneumonia or atelectasis) and present a collection of sample instances. These programs serve as effective teaching aids for medical students, especially when equipped with a question-and-answer module. Establishing an infrastructure for numerous applications is a more systematic approach. The infrastructure's objective should be to construct a repository of images and image-related data that can be utilized across various instructional contexts. Combined with course-authoring tools, such a system could serve as an excellent didactic teaching aid. Alternatively, a free-form browser could be linked to the source structure for a less structured instructional tool. This approach enables students to navigate the system, delving deeper into topics of interest while bypassing less significant ones. Furthermore, the infrastructure could be a foundation for decision-support systems known as "knowledge servants" [9-11].

Assuring the availability of appropriate test cases is one of the biggest challenges in medical imaging research. Several hundred test cases are often required to verify or refute the null hypothesis conclusively. For logistical reasons, test subjects (radiologists) and test cases are typically selected from the same institution. However, differences between clinical and research readings can lead to a significant "memory effect" issue. A PACS can prove invaluable in such scenarios by establishing connectivity among participating institutions. Consequently, institutions gain access to sister institutions' PACS archives and can transfer relevant cases for their studies.

PACS connectivity facilitates multi-institutional research collaboration as an extension of this concept. Such studies are particularly crucial for evaluating clinical efficacy, as even minor changes may require a large number of instances to demonstrate. Since interinstitutional variation is often a concern, multi-institutional studies gain additional relevance when conducting cost-effectiveness analyses of a PACS.

Efficient and rapid image data transfer between capture, archive, and display stations is critical in any PACS configuration. Although dedicated point-to-point physical connections give the finest performance, they are challenging to deploy in a cost-effective manner. These relationships are typically restricted to particular geographical regions. This precise point-to-point connection was a vital component of the American College of Radiology (Reston, VA) and National Electrical Manufacturers Association (Rosslyn, VA) (ACR-NEMA) standard, versions 1 and 2, often known as the 5041 ACR-NEMA connectors [12-15].

Results

PACS has brought about a significant change in the field of radiology, impacting research and education.

Transmission Media

Transmission media are the physical channels that convey data between the nodes in a network. Although wire and cable are extensively utilized, additional wireless communication methods include radio frequencies for short-distance networks and terrestrial and satellite microwaves for long-distance networks. These media are utilized for more than simply data transmission; they are also employed for media rendering. *Coaxial Cable*

The coaxial cable comprises a core conductor wrapped by a tube insulator and an outer shield conductor. For several years, this medium held favor for high-speed network connections owing to its quick transmission speeds, reduced sensitivity to electromagnetic interference, and cost-effectiveness. Despite its extensive use, a noteworthy downside is the necessity for subterranean or overhead installation for signal transmission and receiving, among other functions.

Wireless

As a medium for data transfer, wireless interconnectivity utilizes radio frequency transmission, enabling the transmission and reception of signals through radio frequencies and other wireless mediums. The widespread adoption of these mediums is increasingly evident and holds immense promise for the future of communications. Although undergoing significant development, their universal acceptance is hindered by cost and technological constraints.

PACS Workstations

PACS workstations and displays offer novel capabilities, allowing users to view images innovatively, manipulate images, facilitate computer-assisted diagnosis, and share images across multiple locations. To garner acceptance among clinicians and radiologists for soft-copy displays, these systems must be tailored to meet user requirements, with outcomes varying depending on specific usage scenarios.

Three primary locations requiring PACS workstations include remote sites necessitating image reviews, such as intensive care units or clinicians' offices; workstations for advanced image processing, encompassing three-dimensional rendering, image fusion, and color enhancement; and workstations for the diagnostic interpretation of high-resolution grayscale images (e.g., digitized film) and grayscale multi-image studies (e.g., CT and MRI). Additionally, a fourth type of PACS workstation serves for system administration tasks, routing analyses, and PACS computer control without the need for high-quality image presentation. It is essential for a PACS workstation to handle various images from diverse sources effectively. However, a workstation lacking the operator's workspace is not classified as a PACS workstation by this criterion [16-20].

Researchers and developers have extensively recorded the creation and evaluation of workstations. The following sections examine the physical qualities and functional capabilities of conventional radiography workstations, highlighted by brief examples of three workstation configurations.

Workstation by Trionix

Trionix's computer station offers a sophisticated program allowing for three-dimensional visualization, region of interest measurements, creation of functional studies, back projection of single-photon emission computerized tomography (SPECT) data, and reprojection of SPECT slice data. The incorporation of color is deemed beneficial for assessing functional images, as it can convey pixel intensity or a range of intensities. Despite its complexity, technologists and radiologists in New Mexico have embraced the Sunview interface. These workstations are the hub for initial diagnoses, comparison with previous research, and reprocessing nuclear medicine data for studies. Despite the interface's intricacy, these workstations have been used for three years and garnered widespread acceptance. Notably, they do not require any specialized hardware.

Digital Diagnosis Station (DDS) software (ISG Technologies, Overland Park, KS) is built on an object-oriented image application development environment compatible with various UNIX platforms. It supports multiple color or grayscale 1k monitors and offers a user-configurable environment for displaying and processing images at various levels of complexity, from simple techniques like single-planar reconstruction to more intricate ones like displaying 3-D objects imported from the Allegro system by ISG Technologies. Multiple image studies for a patient can be examined simultaneously through vertical film strips that can be easily scrolled up and down to view corresponding slices from different investigations. A test software program facilitates multisensory fusion or the superimposition of multiple images. The commendable user interface ensures ease of use, and the software benefits from running on a faster workstation, leveraging flexible hardware.

Using PACS workstations and displays, users can explore images in innovative ways, conduct image manipulation and computer-assisted diagnosis, and share images across multiple locations. However, clinicians and radiologists must cater to user requirements to embrace soft-copy displays, with outcomes varying depending on specific usage scenarios.

Medical Imaging Gateway

Currently, this cluster oversees picture acquisition from digital devices and ultrasound, archives all images, and dispatches them to workstations for review or diagnostic interpretation in abdominal radiology, neuroradiology, pediatric radiology, and musculoskeletal radiology. Adding more workstations could expand the system to encompass various radiology clinical clusters. An optical jukebox serves as the archive, where CT and MRI pictures are transferred via a medical imaging gateway (MIG; Vortech Engineering, Channel Islands, CA). MRI scans from GE HealthCare (Chicago, IL) vs the 5× unit communicate images to the MIG over transmission control protocol/Internet protocol (TCP/IP), facilitated by a server specifically designed for this purpose called the Digital Imaging and Communications in Medicine (DICOM) toolkit (DeJarnette Research Systems, Towson, MD ). Images from the Magnatom MR device (Siemens Healthineers, Erlangen, Germany) are transferred via DECnet protocols over TCP/IP. By configuring Vortech's MIG to appear to the Magnatom as an additional MR or CT unit and copying images between working spaces via Siemens MR/CTnet, the transfer is accomplished. This process requires technician assistance but is initiated from the operator's console. A DICOM server, currently under clinical testing, will soon replace MR/CTnet. Images from the two GE advantage CT instruments are sent to the MIG via GE's Network Interface Equipment (NIE) board. AGFA HealthCare (Mortsel, Belgium) uses a frame grabber to convert US pictures from analog to digital DICOM format. The images are then directly connected to AGFA's MG-1000 gateway, where optical character recognition software extracts patient information from the image and generates a DICOM header [21-26].

A Sun 41470 computer (Sun Microsystems, Santa Clara, CA) with functional storage, an operating system, and PACS software on a 3-GB online magnetic disc serves as the foundation of the MIG. The numerous complex tasks of collecting, archiving, database management, jukebox management and control, user interface management, and retrievals became apparent after the initial installation. PACS implementation procedures were delegated to three MIGs, including the original Sun 4/470 and two Sun SPARCstation 2 systems. The Sun 4/470 was assigned the acquisition duty due to its possession of the Versa Module Europa bus required to connect to the CT unit's NIE through a 50-pin ACR-NEMA 2.0 connection. Three gigabytes of the magnetic disc was allocated as an acquisition buffer to retain photographs for at least a day in case of archive failure, ensuring continued system operation while any archive issues are resolved. Images are transferred to workstations for immediate use by technologists and for archiving purposes. One SPARCstation 2 manages user interface connections and workstation interfaces. Study retrieval requests are processed in the user interface, and lists are issued using ACR-NEMA 2.0. The PACS databases, Sybase database management system, jukebox, and 12 GB of magnetic working storage are handled by the other SPARCstation 2, facilitating quicker retrieval of recent studies. Ethernet running TCP/IP facilitates communication between the three MIGs. Digital radiology images are stored on a 100-platter 1C inch and optical platter jukebox manufactured by Kodak (Rochester, NY). The workstations used for digital radiology viewing consist of three personal display systems by Kodak based on the Macintosh and one DDS by ISG Technologies. Radiologists are deemed to require a high-resolution (2k × 2k) display for primary diagnosis. An internal workstation with Mega-Scan displays is currently under construction to fulfill this need.

A hands-on quality control program has been established for PACS components. Each day, display stations are verified by sending an image or group of images to each one to ensure correct operation, display a spatial resolution test pattern, and perform any necessary housekeeping (cleaning the area, the mouse, and the trackball, if applicable). The teleradiology system for the intensive care unit also tests the digitizer. Testing and routine maintenance of the display stations are assigned to a service engineer who also conducts minor repairs on other radiography equipment. All activities are recorded in a logbook. This engineer serves as the first line of defense in case of a failure, classifying the problem and acting as a point of contact for the manufacturer's service division. The quality control process will soon include brightness measurements to detect monitor problems and help anticipate the need for monitor replacement. The digital radiology PACS is automatically checked every 15 minutes by a mechanism that also verifies the functionality of other systems and operations. When a problem is identified, a pager message with the problem's classification and an email message are sent to the system administrator. The caller can then use the network or dial in to perform further repairs and diagnostics. Every 30 minutes, database transaction logs are backed up to recorder tape, and the full PACS database is automatically backed up daily. System issues are reported via email and phone to the vendor and the entire system team. System defects are defined as problems that do not lead to system failure. A continuous status log is maintained using weekly email message sorting [27-30].

Impact of PACS in ICU

To get a deeper comprehension of how PACS impacts efficiency, decision-making, and communication practices in the ICU, as well as its consequences for patient care, it is recommended that future research concentrates on enhancing the outcome measures employed in existing studies and devising novel objective indicator measures. Implementing PACS and its associated technologies will influence and alter work practices in the ICU, depending on the specific setting and organizational structure. Love et al.'s study of the literature on the provider order input in the ICU environment found a "lack of convincing evidence showing its worth" and stressed the importance of employing a sociotechnical approach for future assessments. These findings are also applicable to PACS, and future research should consider the ICU environment while developing and enhancing these indicators. Nevertheless, due to the extensive use of PACS in numerous ICUs, doing any pre- and postresearch is expected to provide significant difficulties. Therefore, the initial research mentioned in this review provides valuable reference points for ICU pre-PACS experiences, which can assist in understanding future discoveries [31-34].

## Conclusions

The PACS represents a versatile tool capable of serving educational and research objectives, offering a plethora of intriguing opportunities. Nonetheless, realizing these objectives necessitates developing and implementing a reliable system for coding medical information associated with films. From an engineering perspective, a meticulously designed interface is imperative to facilitate seamless information exchange among digital imaging devices. Furthermore, recent advancements in high-speed metropolitan area networking are poised to provide significant advantages for integrating institutions across various locations.
